# Knowledge, attitudes, and practices of pharmacists on hierarchical management of COPD: a cross-sectional study in Jiangsu Province

**DOI:** 10.3389/fmed.2025.1616768

**Published:** 2025-06-27

**Authors:** Qiuhui Wu, Yanhong Meng, Ruobin Zhang, Yuhang Ding, Yongsheng Wang, Jinping Zhang

**Affiliations:** ^1^Department of Pharmacy, Nanjing Drum Tower Hospital, Affiliated Hospital of Medical School, Nanjing University, Nanjing, China; ^2^Department of Pharmacy, Nanjing Drum Tower Hospital, School of Basic Medicine and Clinical Pharmacy, China Pharmaceutical University, Nanjing, China; ^3^Department of Respiratory and Critical Care Medicine, Nanjing Drum Tower Hospital, Affiliated Hospital of Medical School, Nanjing University, Nanjing, Jiangsu Province, China

**Keywords:** pharmacists, chronic obstructive pulmonary disease, hierarchical management, KAP, survey

## Abstract

**Purpose:**

This study assessed pharmacists’ knowledge, attitudes, and practices regarding hierarchical management of COPD patients, examining influencing factors to inform standardization.

**Patients and methods:**

A cross-sectional survey was conducted from December 2022 to March 2023 using convenience sampling, yielding 206 valid responses via WeChat-distributed questionnaires. Data analysis employed t-tests, ANOVA, and multiple linear regression.

**Results:**

Pharmacists’ mean scores were: knowledge (6.13 ± 1.96), attitudes (27.56 ± 3.37), and practices (21.02 ± 6.06). Regression analysis highlighted correlations between knowledge scores and gender, education, and hospital level (*p* < 0.05); attitudes and academic qualifications (*p* < 0.05); practices and hospital level (*p* < 0.05).

**Conclusion:**

Pharmacists demonstrated moderate knowledge, positive attitudes, and average practices in COPD management. Tailored training programs addressing educational and institutional disparities are essential to enhance their capacity in hierarchical COPD care.

## 1 Introduction

Chronic obstructive pulmonary disease (COPD), a preventable respiratory condition characterized by persistent airflow limitation (1), is the third leading cause of death worldwide (2). In China, COPD prevalence has surged due to aging populations and environmental factors (2), intensifying the demand for evidence-based management strategies. Pharmacists, as key members of multidisciplinary teams, are increasingly expected to adopt patient-centered approaches aligned with international guidelines such as the Global Initiative for Chronic Obstructive Lung Disease (GOLD) (3).

In recent years, healthcare reforms in China have redefined the role of pharmacists from traditional dispensing to patient-centered care, emphasizing hierarchical management (i.e., stratifying patients by disease severity and medication complexity) as outlined in the Standards for Pharmaceutical Services in Medical Institutions (2019) (4). This policy aligns with GOLD recommendations for personalized COPD care (3), aiming to optimize pharmaceutical service efficiency and patient outcomes.

However, despite policy progress, barriers such as inconsistent adherence to GOLD guidelines, limited clinical training, and fragmented workflows hinder the implementation of hierarchical COPD management in China. To address this gap, this study applies the Knowledge-Attitude-Practice (KAP) model (5)—a widely used framework in healthcare behavior research—to evaluate pharmacists’ competencies and identify systemic bottlenecks. By analyzing factors such as education level and hospital level, this research aims to inform standardized protocols tailored to China’s diverse healthcare settings.

## 2 Materials and methods

### 2.1 Objects and methods of research

The study was conducted as a cross-sectional survey from December 2022 to March 2023 using a convenience sampling method. The study population comprised pharmacists at all levels of hospitals in Jiangsu Province. A recruitment notice was published on the official platform of the Jiangsu Pharmaceutical Association, and a total of 206 pharmacists participated in this project. Subsequently, the pharmacists proceeded to join the WeChat group by scanning the QR code. The research team published a link to the electronic questionnaire (hosted on the Questionnaire Star platform) in the WeChat group. The researchers employed the Questionnaire Star platform to facilitate the collection of the requisite questionnaires. The respondents completed the questionnaire anonymously and submitted it within a seven-day period. The front page of the questionnaire explicitly articulated the objective of the study, the principle of data anonymity, and the voluntariness of participation. The act of submitting the questionnaire was considered a form of informed consent.

### 2.2 Questionnaire

The “Questionnaire on COPD Pharmacists’ Ability and Willingness to Manage Hierarchically” was designed based on the theory of Knowledge, Attitude, and Practice (KAP) (5). The questionnaire is comprised of 32 questions which are divided into four parts:

The initial section of the study comprised a demographic component, encompassing the pharmacist’s gender, age, highest level of education, professional designation, current hospital and job status, and years of experience. Additionally, the pharmacists were queried about the factors that impeded their ability to undertake hierarchical management of COPD patients.

The second part is mainly to understand the pharmacists’ knowledge of COPD patient classification and management, based on the 2024 GOLD Guidelines for COPD disease treatment and management strategies (3), to determine the specific content of the COPD knowledge questionnaire for the pharmacists, covering the disease diagnosis of COPD, characteristics, risk factors, assessment, pulmonary function classification and GOLD grouping and other aspects. There were 10 entries for this dimension, including seven single-choice and three multiple-choice, with each question scoring 1 point for correctness, no points for errors, and no points for multiple-choice questions with multiple choices, wrong choices, or omissions out of 10 points.

The third section of the questionnaire assessed pharmacists’ attitudes toward hierarchical management of COPD patients through six domain-specific items. Each items was scored using the Likert scale ranging from one point for “strongly disagree” to five points for “strongly agree.” The negatively worded question was reverse coded. The overall score was computed by summing all points, with a maximum achievable score of 30. A higherscore suggested a more positive attitude toward the management.

The fourth part evaluated pharmacists’ practices related to the provision of hierarchical management of patients with COPD, which consisted of six items, with a Likert scale (“never,” “rarely,” “sometimes,” “usually,” and “always”) ranging from one point for “never” to five points for “always.” The total score was computed by adding up all points, with a maximum achievable score of 30.

The Cronbach’s alpha coefficient for the questionnaire was 0.905, indicating high internal consistency. During the questionnaire design process, existing literature and well-established questionnaires were extensively consulted to ensure that the content was both sound and scientific. Furthermore, content validity was appraised by a panel of five experts with expertise in clinical practice, education, and policy. Two rounds of iterative review were conducted, and revisions were based on I-CVI thresholds and qualitative feedback (6). The content validity index (CVI) at the questionnaire level was 0.85, which can be considered a scale with sufficient content validity. The combination of these high reliability and validity indices ensures that the questionnaire is a powerful tool for assessing pharmacists’ knowledge, attitudes, and practices in the hierarchical management of COPD.

### 2.3 Sample size calculation

According to the principle of sample size calculation in cross-sectional studies, the sample size of the survey is 5 ~ 10 times the total entries of the questionnaire (7), and the sample size was increased by 10% to accommodate potential non-responses or data loss The questionnaire used in this study consists of 22 entries, and after calculation, the required sample size was calculated to be 121–242 cases.

### 2.4 Statistical analysis

Data collected via the Questionnaire Star platform were cleaned, coded, and imported into SPSS 26.0 for analysis. Normality of continuous variables was assessed using Q-Q plots, which indicated approximate normal distribution. Categorical variables (such as gender, age, and educational title) were summarized as frequencies and percentages, while normally distributed quantitative variables were expressed as mean ± standard deviation. Variables with potential associations were first screened through univariate analysis (independent t-tests for binary variables and ANOVA for multi-level categorical variables). Variables achieving a significance level of *p* < 0.05 in univariate analyses were subsequently entered into the multiple linear regression model to identify independent predictors of pharmacists’ knowledge, attitudes, and practices. Statistical significance was set at *p* < 0.05.

### 2.5 Ethics approval

The Ethics Committee of the Nanjing Drum Tower Hospital approved this study under the approval number “2025–0221-01.” The study complied with the Declaration of Helsinki. Informed consent was obtained from the participants.

## 3 Results

### 3.1 Demographic characteristics of the participants

A total of 206 pharmacists participated in the survey, with a 100% response rate. As detailed in Table 1, the majority were female (76.7%), aged 30–39 years (51.5%), and held a bachelor’s degree in pharmacy (63.1%). Most worked in tertiary hospitals (70.4%) or community health centers (25.2%), primarily in clinical pharmacy (45.6%) or drug dispensing roles (31.6%). Approximately half had received pharmacy service training (56.3%), and 56.8% practiced in hospitals with dedicated COPD/asthma clinics.

**Table 1:** Demographic characteristics and KAP scores.

Characteristics	N(%)	Knowledge^a^	*p*-value	Attitude^b^	*p*-value	Practice^c^	*p*-value
Total	206	6.13 ± 1.96		27.56 ± 3.37		21.02 ± 6.06	
Gender							
Male	48 (23.3)	5.44 ± 2.34	0.016	27.33 ± 4.23	0.598	22.04 ± 5.94	0.183
Female	158 (76.7)	6.34 ± 1.79		27.63 ± 3.07		20.71 ± 6.08	
Age (years)							
20–29	26 (12.6)	5.69 ± 2.36	0.148	27.00 ± 5.55	0.367	18.73 ± 6.69	0.034
30–39	106 (51.5)	5.96 ± 1.88		27.93 ± 2.99		20.62 ± 5.85	
40–49	61 (29.6)	6.48 ± 1.91		27.33 ± 2.88		22.13 ± 6.10	
>50	13 (6.3)	6.77 ± 1.83		26.69 ± 2.63		23.62 ± 4.81	
Highest degree							
Associate degree	4 (1.9)	2.50 ± 2.65	<0.001	21.00 ± 11.49	<0.001	17.00 ± 11.02	0.092
Bachelor’s degree	130 (63.1)	5.83 ± 2.03		27.35 ± 3.15		20.52 ± 6.18	
Master’s degree	66 (32.0)	6.89 ± 1.35		28.32 ± 2.49		22.39 ± 5.44	
PhD	6 (2.9)	6.67 ± 1.75		28.17 ± 2.23		19.50 ± 4.04	
Professional title							
Primary title	46 (22.3)	5.11 ± 2.24	<0.001	26.80 ± 5.03	0.197	18.89 ± 6.73	0.011
Intermediate title	84 (40.8)	6.08 ± 1.84		28.10 ± 2.71		21.31 ± 6.12	
Associate senior title	56 (27.2)	6.86 ± 1.66		27.50 ± 2.59		21.25 ± 5.42	
High professional title	20 (9.7)	6.65 ± 1.60		27.20 ± 2.88		24.05 ± 4.42	
Hospital level							
Tertiary hospital	145 (70.4)	6.68 ± 1.50	0.000	28.08 ± 2.47	0.001	22.19 ± 5.28	<0.001
Secondary hospital	9 (4.4)	6.44 ± 0.88		27.44 ± 2.40		18.00 ± 4.33	
Community Health Service Centre	52 (25.2)	4.56 ± 2.36		26.14 ± 4.96		18.29 ± 7.27	
Workplace							
Pharmaceutical Dispensing and Supply	65 (31.6)	4.92 ± 2.22	<0.001	26.57 ± 4.62	0.004	19.14 ± 7.04	0.010
Clinical pharmacist	94 (45.6)	6.77 ± 1.39		28.33 ± 2.40		21.86 ± 5.12	
Medicines management	47 (22.8)	6.53 ± 1.84		27.38 ± 2.56		21.94 ± 5.87	
Working experience (years)							
<5	21 (10.2)	5.90 ± 2.19	0.532	27.76 ± 5.37	0.490	20.00 ± 6.91	0.187
5–10	63 (30.6)	5.97 ± 1.97		27.78 ± 3.44		21.08 ± 5.84	
11–20	77 (37.4)	6.12 ± 1.77		27.73 ± 2.86		20.31 ± 5.82	
>20	45 (21.8)	6.49 ± 2.17		26.87 ± 2.88		22.62 ± 6.22	
Pharmacy services training							
Yes	116 (56.3)	6.28 ± 1.93	0.203	27.60 ± 2.89	0.828	21.85 ± 5.51	0.026
NO	90 (43.7)	5.93 ± 2.00		27.50 ± 3.91		20.00 ± 6.58	
Pharmacy clinic							
Yes	117 (56.8)	6.56 ± 1.65	<0.001	27.27 ± 3.91	0.284	22.23 ± 5.08	0.001
NO	89 (43.2)	5.56 ± 2.20		19.43 ± 6.86		19.43 ± 6.86	
Hindrance							
Insufficient manpower of pharmacists	173 (84.0)						
Lack of time for patient management	178 (86.4)						
Lack of clear implementation criteria	130 (63.1)						
Lack of specialized workplaces and equipment	134 (65.0)						

Data are presented as n (%) or Mean ± SD.

KAP, knowledge, attitude, and practice.

a Knowledge:10 items, scored 0 or 1 per item (total range:0–10).

b Attitude: 6 Likert-scale items (1–5 points per item; total range: 6–30).

c Practice: 6 Likert-scale items (1–5 points per item; total range: 6–30).

The total scores for pharmacists’ knowledge, attitudes, and practices in hierarchical COPD management ranged from 13 to 70, with a mean of 54.71 ± 8.48. Specifically, the knowledge dimension scored 6.13 ± 1.96 (range 0–10), attitudes scored 27.56 ± 3.37 (range 6–30), and practices scored 21.02 ± 6.06 (range 6–30). These results indicate that pharmacists possess moderate knowledge, positive attitudes, and average practices in hierarchical COPD management. The KAP scores of pharmacists with different demographic characteristics in the hierarchical management of COPD patients were analyzed by single-factor ANOVA (Table 1). The analysis revealed that in addition to job types, factors such as sex, age, education level, professional rank, training participation, and hospital level were found to have significant impacts on the KAP score.

Bivariate analysis, employing independent *t*-tests and single-factor ANOVA, examined the relationship between pharmacists’ KAP scores for graded management of COPD and various social-demographic variables (Table 1). The analysis revealed that in addition to job types, factors such as sex, age, education level, professional rank, training participation, and hospital level were found to have significant associations with the KAP scores.

### 3.2 Pharmacists’ knowledge, attitude, and practice scores for hierarchical management of COPD patients

**Figure 1:**
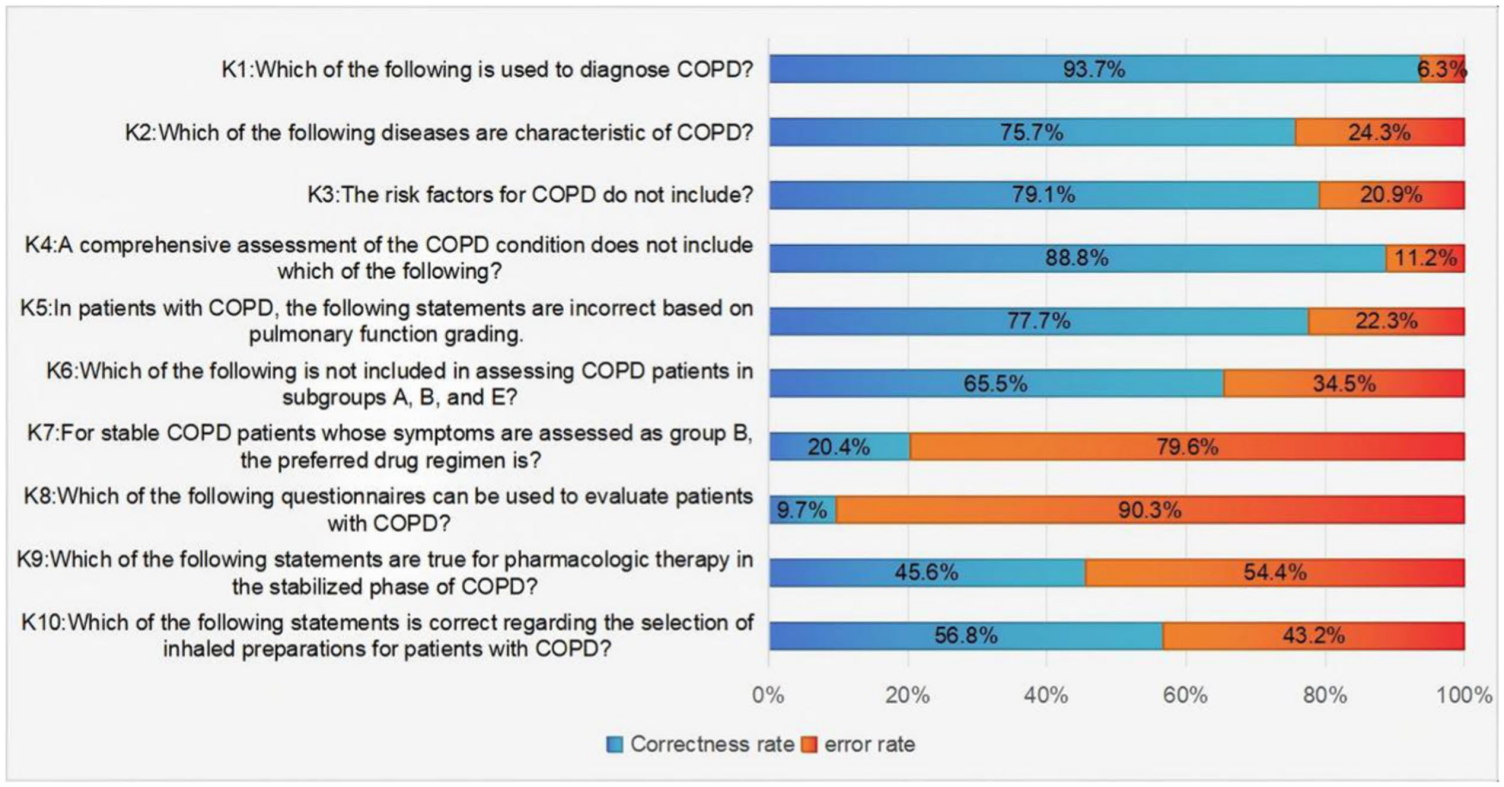
Pharmacists’ answers to knowledge items about the hierarchical management of patients with COPD.

As demonstrated in Figure 1, pharmacists demonstrated strong knowledge in key areas: COPD diagnosis (93.7% correct), COPD risk factor (79.1%), and pulmonary function classification (77.7%). In contrast, the lowest values were observed for the item “Which of the following questionnaires can be used to evaluate patients with COPD?,” with a mere 9.7% of respondents providing a positive response. Furthermore, only 20.4% of pharmacists were aware that the preferred drug regimens for stable COPD patients with symptom assessments in Group B were LABA and LAMA.

**Figure 2:**
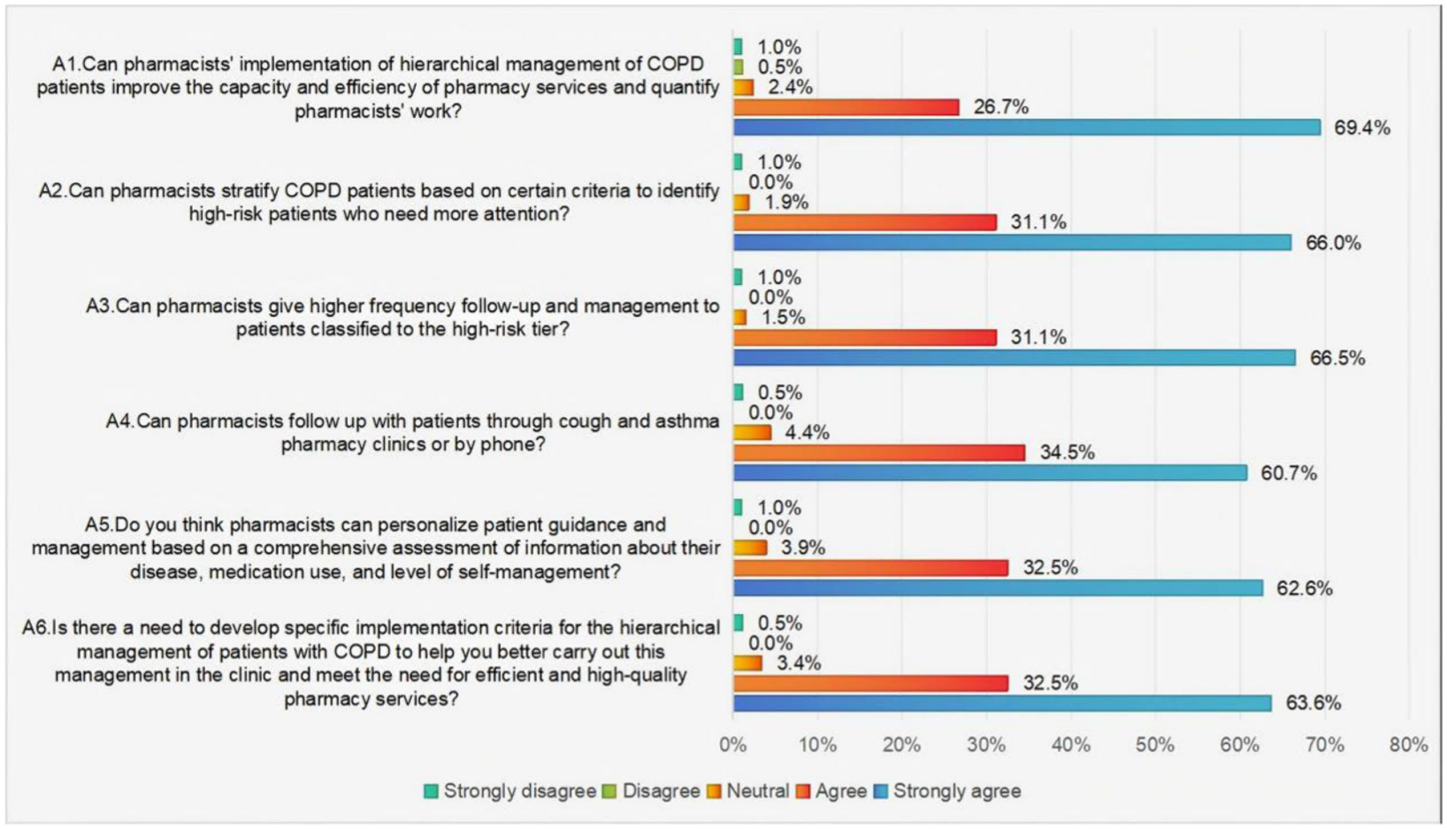
Pharmacists’ responses to items assessing attitudes toward the hierarchical management of patients with COPD.

Figure 2 utilizes a series of six inquiries to assess the prevailing attitudes of pharmacists concerning the implementation of hierarchical management for COPD patients. The aggregate analysis revealed that nearly 90% of pharmacists expressed agreement or strong agreement with the approach of conducting comprehensive assessments, stratification, and providing personalized guidance for COPD patients. Furthermore, a substantial majority of pharmacists (96.1%), expressed concurrence or strong agreement with the notion that the implementation of a hierarchical management system for COPD patients has the potential to augment the capacity and efficiency of pharmacy services, while concurrently enabling the quantification of pharmacists’ contributions. This finding collectively underscores the positive attitudes of pharmacists toward the implementation of the hierarchical management of COPD patients.

**Figure 3:**
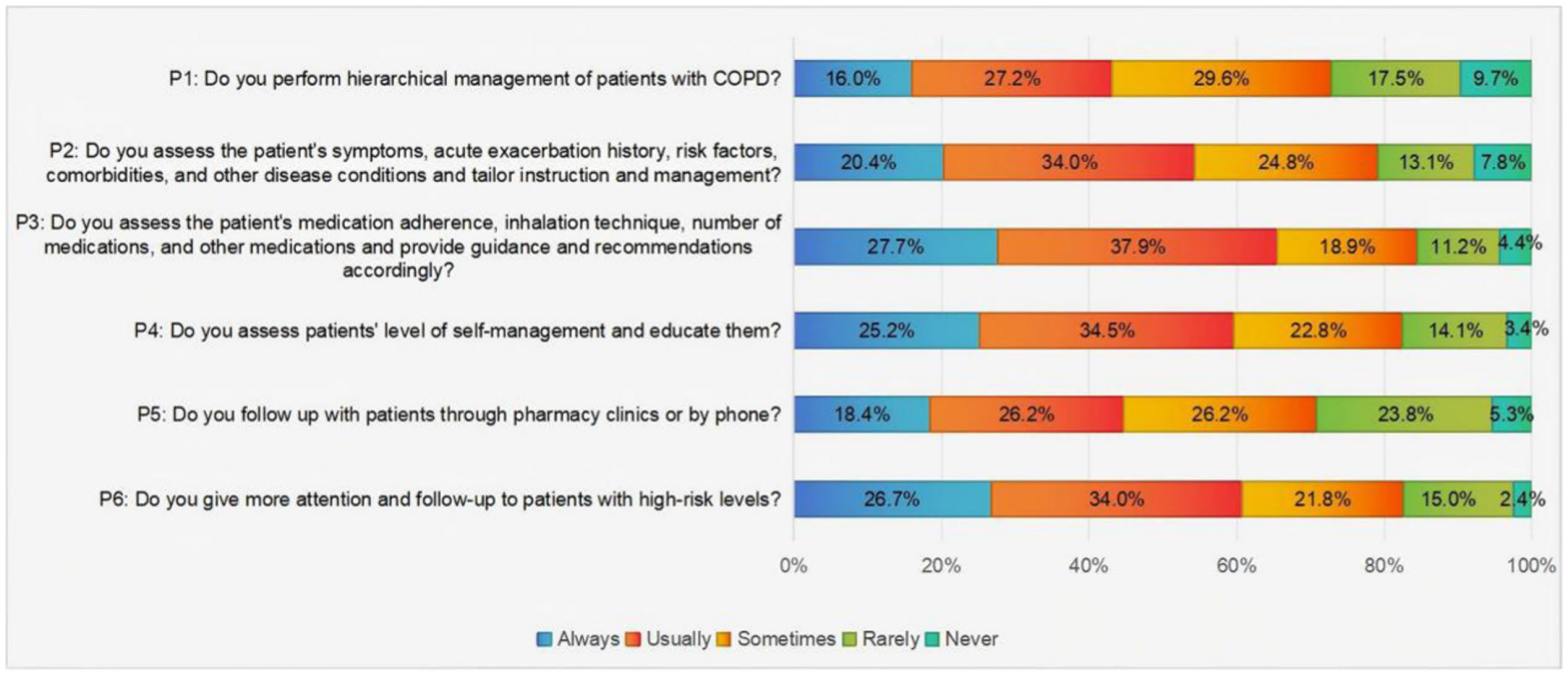
Pharmacists’ responses to the hierarchical management of patients with COPD practice items.

As illustrated in Figure 3, the current state of practice regarding pharmacists’ involvement in COPD management indicates that those who selected the “always” and “usually” options were identified as having successful COPD management practices. A mere 43% of pharmacists can implement hierarchical management of COPD patients and follow up with patients in the clinic or by phone. Meanwhile, approximately 60% of pharmacists frequently focus on high-risk patients, assessing their self-management behaviors and providing them with educational guidance.

### 3.3 Pharmacists’ multifactorial analysis of KAP for hierarchical management of COPD patients

Pearson’s correlation analysis showed positive correlations between pharmacists’ knowledge and attitudes (*r* = 0.191, *p* < 0.01), knowledge and practices (*r* = 0.214, *p* < 0.01), and attitudes and practices (*r* = 0.304, *p* < 0.01) in the hierarchical management of COPD patients.

The three dimensions of KAP in the hierarchical management of COPD patients by pharmacists were used as dependent variables. According to the results of the single-factor ANOVA, the corresponding meaningful demographic characteristics were selected as independent variables (*p* < 0.05). The results of the multiple linear regression analysis are shown in Table 2. The findings of the multifactorial analysis of knowledge demonstrated that gender, education, and hospital level were influential factors affecting pharmacists’ knowledge scores in performing hierarchical management of COPD patients (*p* < 0.05). Female pharmacists demonstrated superior knowledge, while pharmacists with bachelor’s, master’s, and doctoral degrees exhibited higher knowledge scores compared to those with associate degree (*p* < 0.05). Pharmacists working in community health centers exhibited lower knowledge compared to their counterparts in tertiary hospitals (*p* < 0.05). The results of the attitudinal multifactorial analysis indicated that education was a significant predictor of attitude scores (*p* < 0.05). Specifically, pharmacists who possessed a bachelor’s degree or higher exhibited more favorable attitudes toward implementing hierarchical management strategies for COPD patients compared to pharmacists who held an associate degree. The findings of the practice multifactorial analysis indicated that hospital grade was a significant predictor of practice scores (*p* < 0.05). Pharmacists working in community health centers demonstrated significantly lower adherence to evidence-based COPD management protocols compared to pharmacists working in tertiary hospitals.

**Table 2:** Results of multiple linear regression analyses affecting pharmacists’ knowledge, attitude and practice for hierarchical management of COPD patients (*N* = 206).

Model	Variable	β	*t*	95% Cl	*p*-value
Knowledge	(Const)		2.716	(0.717, 4.518)	<0.001
	Gender				
	Male	RC			
	Female	0.165	2.759	(0.217, 1.307)	0.006
	Highest degree				
	Associate degree	RC			
	Bachelor’s degree	0.631	2.962	(0.856, 4.257)	0.003
	Master’s degree	0.681	3.097	(1.037, 4.675)	0.002
	PhD	0.217	2.244	(0.306, 4.744)	0.026
	Professional title				
	Primary title	RC			
	Intermediate title	0.079	0.965	(−0.328, 0.956)	0.336
	Associate senior title	0.171	1.945	(−0.011, 1.520)	0.053
	High professional title	0.048	0.588	(−0.747, 1.382)	0.557
	Hospital level				
	Tertiary hospital	RC			
	Secondary hospital	0.048	0.745	(−0.753, 1.666)	0.457
	Community Health Service Centre	−0.265	−3.141	(−1.941, −0.444)	0.002
	Workplace				
	Pharmaceutical Dispensing and Supply	RC			
	Clinical pharmacist	0.130	1.356	(−0.233, 1.257)	0.177
	Medicines management	0.112	1.309	(−0.265, 1.311)	0.192
	Pharmacy clinic				
	Yes	RC			
	No	−0.066	−0.987	(−0.780, 0.260)	0.325
Attitude	(Const)		12.406	(18.270, 25.176)	
	Highest degree				
	Associate degree	RC			
	Bachelor’s degree	0.831	3.479	(2.505, 9.062)	0.001
	Master’s degree	0.833	3.390	(2.510, 9.489)	0.001
	PhD	0.292	2.688	(1.511, 10.098)	0.008
	Hospital level				
	Tertiary hospital	RC			
	Secondary hospital	0.015	0.212	(−2.022, 2.511)	0.832
	Community Health Service Centre	−0.142	−1.622	(−2.427, 0.236)	0.106
	Workplace				
	Pharmaceutical Dispensing and Supply	RC			
	Clinical pharmacist	0.106	1.001	(−0.692, 2.119)	0.318
	Medicines management	0.019	0.224	(−1.185, 1.488)	0.823
Practice	(Const)		12.659	(17.653, 24.169)	
	Age				
	20–29	RC			
	30–39	0.146	1.184	(−1.179, 4.723)	0.238
	40–49	0.235	1.661	(−0.583, 6.811)	0.098
	>50	0.126	1.132	(−2.333, 8.614)	0.259
	Professional title				
	Primary title	RC			
	Intermediate title	0.005	0.049	(−2.580, 2.712)	0.961
	Associate senior title	−0.129	−0.987	(−5.247, 1.745)	0.325
	High professional title	0.000	−0.001	(−4.918, 4.914)	0.999
	Hospital level				
	Tertiary hospital	RC			
	Secondary hospital	−0.095	−1.323	(−6.971, 1.374)	0.188
	Community Health Service Centre	−0.189	−2.002	(−5.226, −0.038)	0.047
	Workplace				
	Pharmaceutical Dispensing and Supply	RC			
	Clinical pharmacist	0.041	0.399	(−1.947, 2.936)	0.690
	Medicines management	0.076	0.776	(−1.692, 3.885)	0.439
	Pharmacy services training				
	Yes	RC			
	No	−0.112	−1.610	(−3.039, 0.307)	0.109
	Pharmacy clinic				
	Yes	RC			
	No	−0.108	−1.405	(−3.156, 0.531)	0.162

β, regression coefficient; CI, confidence interval; RC, reference category.

## 4 Discussion

This study assessed the knowledge, attitudes, and practices of pharmacists in Jiangsu Province, China, with regard to the hierarchical management of COPD patients. The results revealed that the pharmacists exhibited moderate knowledge, positive attitudes, and average practices Jiangsu Province. It was notable that female pharmacists, those with higher educational qualifications, and those working in tertiary hospitals demonstrated superior knowledge scores. Education level was a significant predictor of attitude scores, while hospital level was a crucial predictor of practice scores.

The findings of this study indicated that pharmacists demonstrated average levels of knowledge regarding the hierarchical management of COPD. While the participants demonstrated competencies in key areas such as COPD diagnosis, comprehensive disease assessment protocols, and lung function classification, their knowledge was found to be insufficient concerning the key elements of the GOLD 2024 guideline update, particularly in distinguishing between grouping principles and assessment tools. This finding is consistent with the results of a previous study on Finnish community pharmacists (8, 9), which showed that nearly half were unfamiliar with the current guidelines for the management of COPD. The GOLD 2024 guidelines introduced individualized approaches to patient assessment and treatment that are currently not adequately disseminated and incorporated into pharmacists’ routine training programs (3). The study also revealed a positive driving effect of pharmacists’ hospital level and education on knowledge scores. Pharmacists in tertiary hospitals had higher knowledge scores than pharmacists in community hospitals, and pharmacists with a bachelor’s degree or higher had significantly better knowledge than pharmacists with associate degree. Pharmacists who hold advanced academic degrees, such as a Master’s or Doctoral degree, demonstrated enhanced competencies in the management of COPD. Additionally, these individuals exhibited increased access to information resources and demonstrated a higher level of clinical pharmacy knowledge. A noteworthy finding is the observation that the knowledge scores of pharmacists with master’s degrees exceeded those of pharmacists with doctoral degrees. This discrepancy did not seem to be attributable to a difference in intrinsic competence between the two groups. This discrepancy may be attributed to the fact that pharmacists with master’s degrees tend to engage more extensively in clinical services and patient management throughout their professional trajectories. In contrast, pharmacists with doctoral degrees typically prioritize clinical research upon entering the hospital setting, which may have a direct impact on their performance in terms of clinical knowledge and skills (10). This underscores the significance of knowledge-to-practice transfer, emphasizing the necessity to address the identified knowledge gap through modular training programs (such as “GOLD Core Update Express”), complemented by real-life case simulation training to enhance the practical application skills of Associate degree and community pharmacists (This study specifically refers to the group of pharmacists engaged in primary healthcare services in the primary healthcare system in China). Additionally, the updated content of the GOLD annual guidelines should be incorporated into the continuing education credit system for pharmacists. This would require them to complete a predetermined amount of COPD-specific learning annually and ensure knowledge internalization through practical-oriented assessment mechanisms, such as case analysis reports.

The prevailing attitude among pharmacists toward the hierarchical management of patients with COPD was one of positivity. The vast majority of them believed that comprehensive assessment, graded management, and individualized instruction for these patients were beneficial. This attitude is consistent with the findings of other similar studies (11, 12) and reflects pharmacists’ recognition of their expanded role. Multiple regression analyses of attitudes revealed a positive correlation between educational attainment and attitudes. As educational attainment increased, pharmacists could understand the scientific basis of hierarchical management, resulting in more favorable attitudes (13). This finding further highlights the critical role of knowledge in attitude formation.

Although pharmacists generally held positive attitudes toward the hierarchical management of COPD, their practice scores indicated a discrepancy between theoretical support and practical application. Specifically, only a minority of pharmacists frequently managed patients with COPD on a tiered basis, and the rates of follow-up management were unsatisfactory. The significant impact of hospital level on practice scores reflects current differences in resource allocation and practice environments. In China, the development of pharmacy care in hospitals is still in its infancy and is mainly provided by secondary and tertiary hospitals (14). Tertiary hospitals have advanced equipment, specialized staff, and well-established workflows, making them more suitable for pharmacists to implement hierarchical management. On the contrary, the role of community pharmacists has not yet been fully transformed, and most community pharmacists still focus on drug supply. Community health centers often lack the necessary resources and a clear definition of the pharmacist’s role (15), which prevents pharmacists from translating their knowledge and attitudes into practice. This “attitude-practice divide” emphasizes the importance of addressing the poor practice ability of community pharmacists by optimizing resource allocation, and several studies have shown that the healthcare consortium model enables efficient collaboration between tertiary and community hospitals, facilitates the sinking of tertiary hospitals’ advantageous resources, and improves the utilization of primary healthcare resources (16, 17). Therefore, we can glean insights from the medical consortium model proposed by Verhulst et al. (18) to establish a regional COPD pharmacy service center overseen by tertiary hospitals. This model facilitates the precise allocation of high-quality resources, such as prescription review and medication education, through the teleconsultation system. Additionally, it fosters the development of a continuity pharmacy service network that utilizes the medical consortium’s data-sharing platform to establish a closed-loop management model of “standardized management in tertiary hospitals and case follow-up in community hospitals.” These measures have been demonstrated to be conducive to the strengthening of the primary drug-patient relationship, as well as to the development of a more effective and efficient drug management system. These measures have been shown to enhance communication between patients and pharmacists at the grassroots level, thereby strengthening the effectiveness of that communication. Moreover, these measures have been shown to significantly enhance the practice enthusiasm and initiative of the pharmacist community through the standardized training system.

The present study employs the Knowledge-Attitude-Practice (KAP) model to elucidate the pivotal function of pharmacists in the hierarchical management of (COPD). These findings align with the fundamental premise of the KAP model, which posits that knowledge serves as the foundation for shaping attitudes, thereby significantly impacting practice behaviors (19, 20). The present study identified a substantial correlation (*p* < 0.05) between educational attainment and favorable attitudes, thereby substantiating the notion that the acquisition of knowledge serves as a pivotal catalyst for attitude modification. However, a discrepancy persists between theoretical acceptance and practical implementation, underscoring the systemic impediments that prove challenging to surmount through reliance on the KAP model alone. The present study underscores the significance of implementing a multilevel intervention strategy to effectively augment the role of pharmacists in the hierarchical management of COPD. This strategy entails enhancing pharmacists’ professional knowledge through enhanced training, ensuring optimal practice behaviors with policy support, and optimizing resource allocation (e.g., through the adoption of the medical consortium model). These measures are designed to enhance the efficacy of pharmacists in the hierarchical management of COPD in a comprehensive manner.

### 4.1 Limitations

The generalizability of the findings may be limited by the study’s exclusive focus on Jiangsu Province, which constitutes a key limitation of this research. Future research should expand the sample size to include more regions, to obtain a more comprehensive understanding of pharmacists’ KAP regarding COPD hierarchical management. Additionally, the cross-sectional nature of the study precludes the determination of causality. Longitudinal studies and qualitative research could provide deeper insights into the motivations and barriers influencing pharmacists’ behaviors, as well as the long-term impacts of educational and policy interventions on their KAP and patient outcomes.

## 5 Conclusion

This study underscores the pivotal role of gender, educational background, and hospital tier in determining pharmacists’ proficiency in COPD hierarchical management. The identified “attitude-practice divide” necessitates targeted educational, policy, and resource interventions to empower pharmacists and enhance the quality of COPD care. By addressing these factors, we can transform pharmacists into more effective core players in chronic disease management, contributing to the global efforts in preventing and controlling chronic respiratory diseases.

## Data Availability

The original contributions presented in the study are included in the article/Supplementary material, further inquiries can be directed to the corresponding authors.
