# Supplementary material for: Knowledge, attitudes, and practices of pharmacists on hierarchical management of COPD: a cross-sectional study in Jiangsu Province
Source: Front Med (Lausanne). 2025 Jun 27;12:1616768. doi: 10.3389/fmed.2025.1616768 (PMC12247054; doi:10.3389/fmed.2025.1616768)
Supplement: Supplementary file 1 [file Table_1.docx]

Supplementary Material

# Supplementary Figures and Tables

## Supplementary Figures


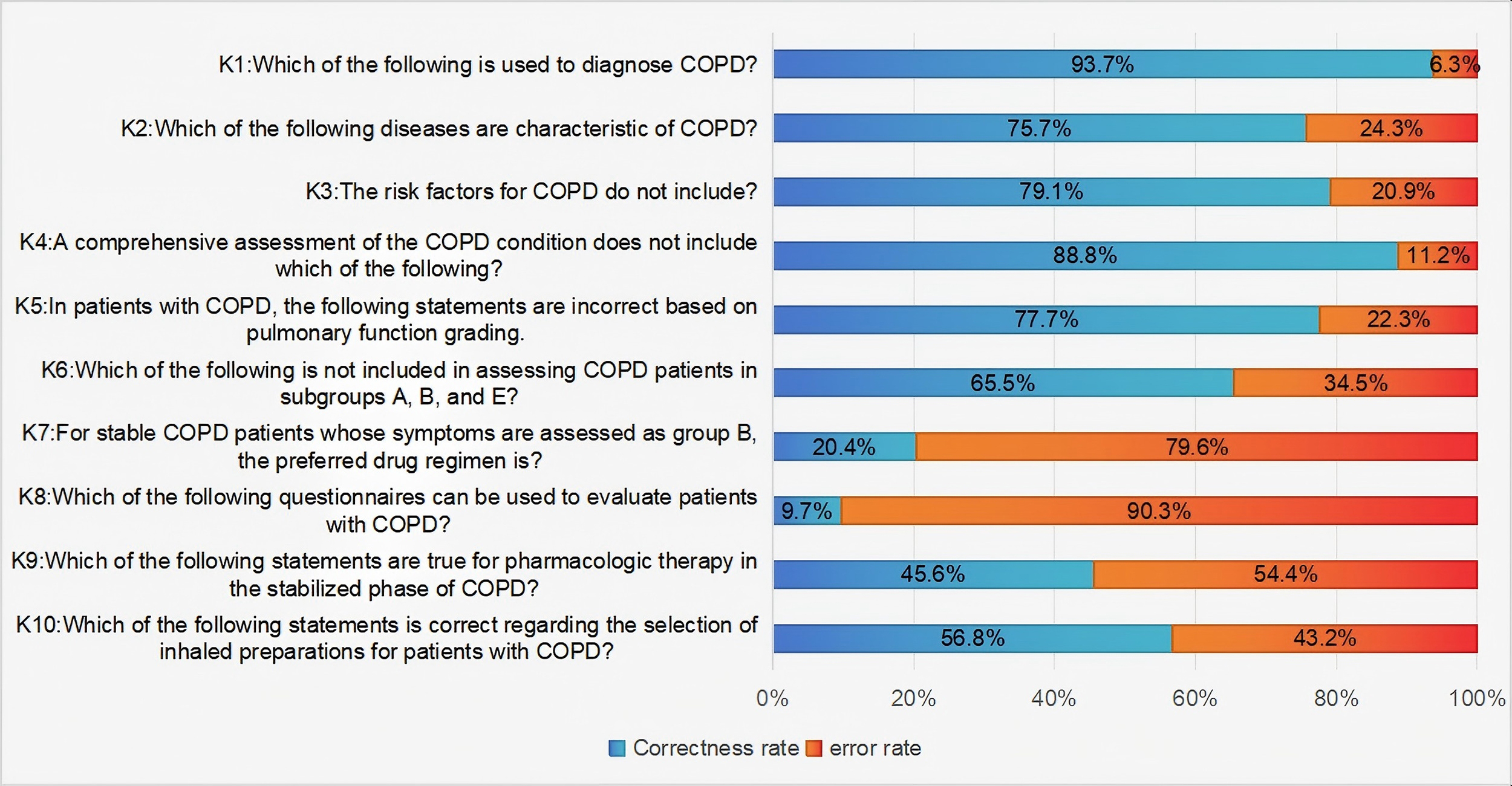


**Figure 1:** Pharmacists' answers to knowledge questions about the hierarchical management of patients with COPD.


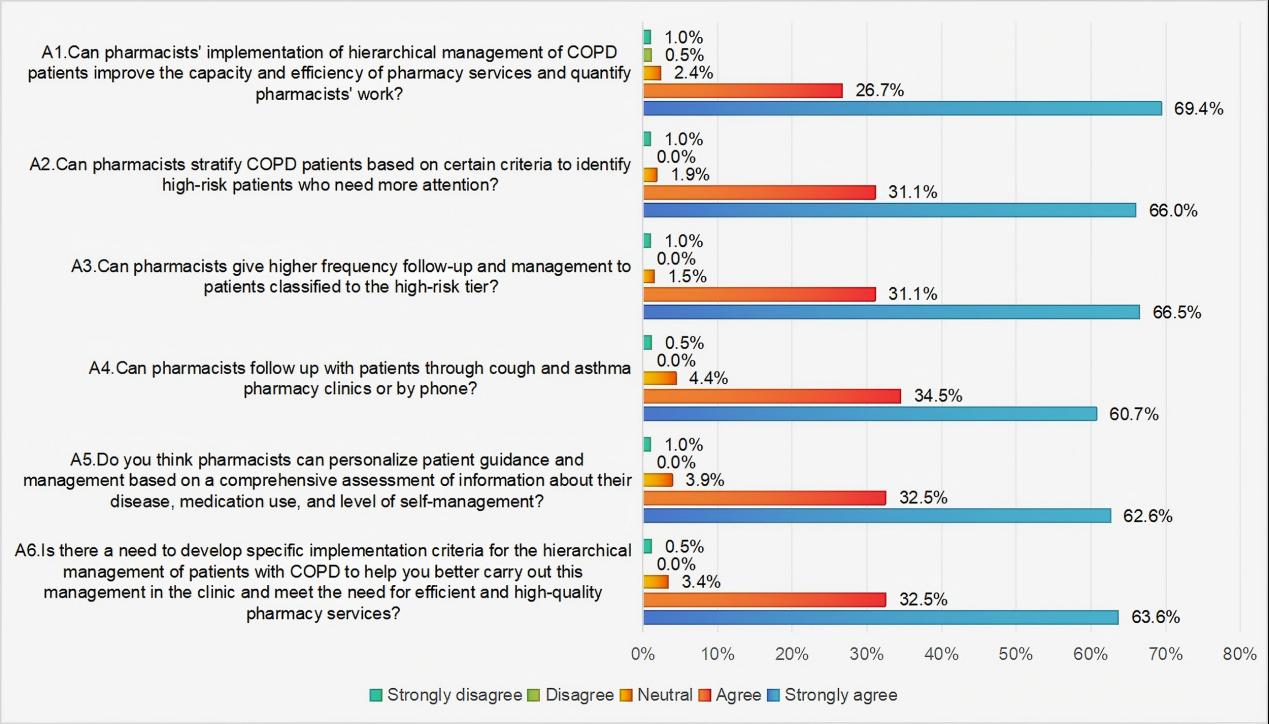


**Figure 2:** Pharmacists' responses to items assessing attitudes toward the hierarchical management of patients with COPD.


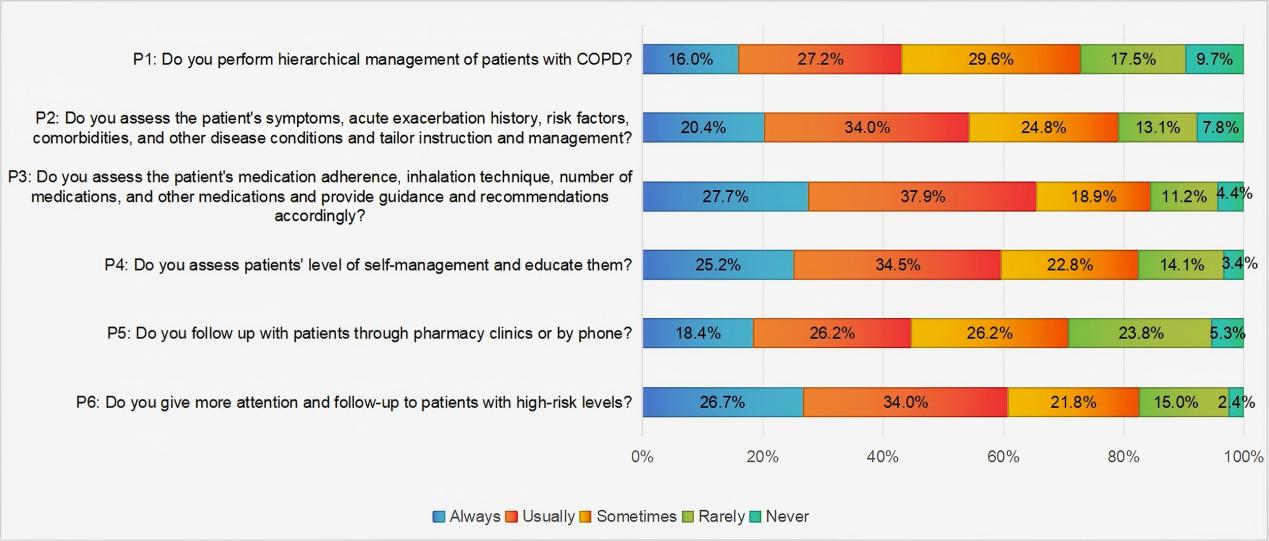


**Figure 3:** Pharmacists' responses to the hierarchical management of patients with COPD practice items.

## Supplementary Tables

**Table 1** Demographic characteristics and KAP scores

| Characteristics | N（%） | Knowledge | *P-value* | Attitude | *P-value* | Practice | *P-value* |
| --- | --- | --- | --- | --- | --- | --- | --- |
| Total | 206 | 6.13±1.96 |  | 27.56±3.37 |  | 21.02±6.06 |  |
| Gender |  | | | | | | |
| Male | 48（23.3） | 5.44±2.34 | 0.016 | 27.33±4.23 | 0.598 | 22.04±5.94 | 0.183 |
| Female | 158（76.7） | 6.34±1.79 |  | 27.63±3.07 |  | 20.71±6.08 |  |
| Age (years) |  | | | | | | |
| 20-29 | 26（12.6） | 5.69±2.36 | 0.148 | 27.00±5.55 | 0.367 | 18.73±6.69 | 0.034 |
| 30-39 | 106（51.5） | 5.96±1.88 |  | 27.93±2.99 |  | 20.62±5.85 |  |
| 40-49 | 61（29.6） | 6.48±1.91 |  | 27.33±2.88 |  | 22.13±6.10 |  |
| >50 | 13（6.3） | 6.77±1.83 |  | 26.69±2.63 |  | 23.62±4.81 |  |
| Highest degree |  | | | | | | |
| Lower than Bachelor’s degree | 4（1.9） | 2.50±2.65 | <0.001 | 21.00±11.49 | <0.001 | 17.00±11.02 | 0.092 |
| Bachelor's degree | 130（63.1） | 5.83±2.03 |  | 27.35±3.15 |  | 20.52±6.18 |  |
| Master's degree | 66（32.0） | 6.89±1.35 |  | 28.32±2.49 |  | 22.39±5.44 |  |
| PhD | 6（2.9） | 6.67±1.75 |  | 28.17±2.23 |  | 19.50±4.04 |  |
| Professional title |  | | | | | | |
| Primary title | 46（22.3） | 5.11±2.24 | <0.001 | 26.80±5.03 | 0.197 | 18.89±6.73 | 0.011 |
| Intermediate title | 84（40.8） | 6.08±1.84 |  | 28.10±2.71 |  | 21.31±6.12 |  |
| Associate senior title | 56（27.2） | 6.86±1.66 |  | 27.50±2.59 |  | 21.25±5.42 |  |
| High professional title | 20（9.7） | 6.65±1.60 |  | 27.20±2.88 |  | 24.05±4.42 |  |
| Hospital level |  | | | | | | |
| Tertiary hospital | 145（70.4） | 6.68±1.50 | 0.000 | 28.08±2.47 | 0.001 | 22.19±5.28 | <0.001 |
| Secondary hospital | 9（4.4） | 6.44±0.88 |  | 27.44±2.40 |  | 18.00±4.33 |  |
| Community Health Service Centre | 52（25.2） | 4.56±2.36 |  | 26.14±4.96 |  | 18.29±7.27 |  |
| Workplace |  | | | | | | |
| Pharmaceutical Dispensing and Supply | 65（31.6） | 4.92±2.22 | <0.001 | 26.57±4.62 | 0.004 | 19.14±7.04 | 0.010 |
| Clinical pharmacist | 94（45.6） | 6.77±1.39 |  | 28.33±2.40 |  | 21.86±5.12 |  |
| Medicines management | 47（22.8） | 6.53±1.84 |  | 27.38±2.56 |  | 21.94±5.87 |  |
| Working experience（years） |  | | | | | | |
| <5 | 21（10.2） | 5.90±2.19 | 0.532 | 27.76±5.37 | 0.490 | 20.00±6.91 | 0.187 |
| 5-10 | 63（30.6） | 5.97±1.97 |  | 27.78±3.44 |  | 21.08±5.84 |  |
| 11-20 | 77（37.4） | 6.12±1.77 |  | 27.73±2.86 |  | 20.31±5.82 |  |
| >20 | 45（21.8） | 6.49±2.17 |  | 26.87±2.88 |  | 22.62±6.22 |  |
| Pharmacy services training |  | | | | | | |
| Yes | 116（56.3） | 6.28±1.93 | 0.203 | 27.60±2.89 | 0.828 | 21.85±5.51 | 0.026 |
| NO | 90（43.7） | 5.93±2.00 |  | 27.50±3.91 |  | 20.00±6.58 |  |
| Pharmacy clinic |  | | | | | | |
| Yes | 117（56.8） | 6.56±1.65 | <0.001 | 27.27±3.91 | 0.284 | 22.23±5.08 | 0.001 |
| NO | 89（43.2） | 5.56±2.20 |  | 19.43±6.86 |  | 19.43±6.86 |  |
| hindrance |  | | | | | | |
| Insufficient manpower of pharmacists | 173(84.0) |  |  |  |  |  |  |
| Lack of time for patient management | 178(86.4) |  |  |  |  |  |  |
| Lack of clear implementation criteria | 130(63.1) |  |  |  |  |  |  |
| Lack of specialized workplaces and equipment | 134(65.0) |  |  |  |  |  |  |

Notes:Data are presented as n (%) or Mean ± SD.

Abbreviations:KAP,knowledge, attitude, and practice.

Table 2 Results of multiple linear regression analyses affecting pharmacists' knowledge, attitude and practice for hierarchical management of COPD patients (N=206)

| Model | Variable | β | t | 95% Cl | *P value* |
| --- | --- | --- | --- | --- | --- |
| Knowledge | (const) |  | 2.716 | （0.717,4.518） | <0.001 |
|  | Gender |  | | | |
|  | Male | RC | | | |
|  | Female | 0.165 | 2.759 | （0.217,1.307） | 0.006 |
|  | Highest degree |  | | | |
|  | Lower than Bachelor’s degree | RC | | | |
|  | Bachelor's degree | 0.631 | 2.962 | （0.856,4.257） | 0.003 |
|  | Master's degree | 0.681 | 3.097 | （1.037,4.675） | 0.002 |
|  | PhD | 0.217 | 2.244 | （0.306,4.744） | 0.026 |
|  | Professional title |  | | | |
|  | Primary title | RC | | | |
|  | Intermediate title | 0.079 | 0.965 | （-0.328,0.956） | 0.336 |
|  | Associate senior title | 0.171 | 1.945 | （-0.011,1.520） | 0.053 |
|  | High professional title | 0.048 | 0.588 | （-0.747,1.382） | 0.557 |
|  | Hospital level |  | | | |
|  | Tertiary hospital | RC | | | |
|  | Secondary hospital | 0.048 | 0.745 | （-0.753,1.666） | 0.457 |
|  | Community Health Service Centre | -0.265 | -3.141 | （-1.941,-0.444） | 0.002 |
|  | Workplace |  | | | |
|  | Pharmaceutical Dispensing and Supply | RC | | | |
|  | Clinical pharmacist | 0.130 | 1.356 | （-0.233,1.257） | 0.177 |
|  | Medicines management | 0.112 | 1.309 | （-0.265,1.311） | 0.192 |
|  | Pharmacy clinic |  | | | |
|  | Yes | RC | | | |
|  | NO | -0.066 | -0.987 | （-0.780,0.260） | 0.325 |
| Attitude | (const) |  | 12.406 | （18.270,25.176） |  |
|  | Highest level of education |  | | | |
|  | Lower than Bachelor’s degree | RC | | | |
|  | Bachelor's degree | 0.831 | 3.479 | （2.505,9.062） | 0.001 |
|  | Master's degree | 0.833 | 3.390 | （2.510,9.489） | 0.001 |
|  | PhD | 0.292 | 2.688 | （1.511,10.098） | 0.008 |
|  | Hospital level |  | | | |
|  | Tertiary hospital | RC | | | |
|  | Secondary hospital | 0.015 | 0.212 | （-2.022,2.511） | 0.832 |
|  | Community Health Service Centre | -0.142 | -1.622 | （-2.427,0.236） | 0.106 |
|  | Workplace |  | | | |
|  | Pharmaceutical Dispensing and Supply | RC | | | |
|  | Clinical pharmacist | 0.106 | 1.001 | （-0.692，2.119） | 0.318 |
|  | Medicines management | 0.019 | 0.224 | （-1.185，1.488） | 0.823 |
| Practice | (const) |  | 12.659 | （17.653，24.169） |  |
|  | Age |  | | | |
|  | 20-29 | RC | | | |
|  | 30-39 | 0.146 | 1.184 | （-1.179，4.723） | 0.238 |
|  | 40-49 | 0.235 | 1.661 | （-0.583，6.811） | 0.098 |
|  | >50 | 0.126 | 1.132 | （-2.333，8.614） | 0.259 |
|  | Professional title |  | | | |
|  | Primary title | RC | | | |
|  | Intermediate title | 0.005 | 0.049 | （-2.580,2.712） | 0.961 |
|  | Associate senior title | -0.129 | -0.987 | （-5.247,1.745） | 0.325 |
|  | High professional title | 0.000 | -0.001 | （-4.918,4.914） | 0.999 |
|  | Hospital level |  | | | |
|  | Tertiary hospital | RC | | | |
|  | Secondary hospital | -0.095 | -1.323 | （-6.971,1.374） | 0.188 |
|  | Community Health Service Centre | -0.189 | -2.002 | （-5.226,-0.038） | 0.047 |
|  | Workplace |  | | | |
|  | Pharmaceutical Dispensing and Supply | RC | | | |
|  | Clinical pharmacist | 0.041 | 0.399 | （-1.947,2.936） | 0.690 |
|  | Medicines management | 0.076 | 0.776 | （-1.692,3.885） | 0.439 |
|  | Pharmacy services training |  | | | |
|  | Yes | RC | | | |
|  | NO | -0.112 | -1.610 | （-3.039,0.307） | 0.109 |
|  | Pharmacy clinic |  | | | |
|  | Yes | RC | | | |
|  | NO | -0.108 | -1.405 | （-3.156,0.531） | 0.162 |

Abbreviations: β, regression coefficient; CI,confidence interval;RC,reference category.

# Supplementary Data

## The KAP Questionnaire on Hierarchical Management of patients with COPD

### Ⅰ. Pharmacists' knowledge questionnaire on hierarchical management of patients with COPD

1. **Which of the following is used to diagnose COPD? （Multiple choice）**

FEV1/FVC□(correct answer) FVC□ TLC□ IC□ IC/TLC□

1. **Which of the following diseases are characteristic of COPD?（Multiple choice）**

Positive bronchodilator test□

Cystic fibrosis with airflow limitation□

Bronchiectasis with airflow limitation□

Chronic bronchitis, emphysema with airflow limitation□(correct answer)

Diffuse panfine bronchiolitis with airflow limitation□

1. **The risk factors for COPD do not include?（Multiple choice）**

Smoking□

Infection□

Air pollution□

Occupational dust□

Allergy□ (correct answer)

1. **A comprehensive assessment of the COPD condition does not include which of the following?（Multiple choice）**

Clinical Symptoms□

degree of airflow obstruction□

History of acute exacerbations□

Comorbidities□

Obesity□ (correct answer)

1. **Which of the following statements is incorrect based on the pulmonary function classification of a patient with COPD?（Multiple choice）**

GOLD 4: FEV1 <30% of predicted value□

GOLD 3: FEV1 as a percentage of the expected value is 30%~49%□

GOLD 3: FEV1 as a percentage of predicted value is 30%~70%□(correct answer)

GOLD 2: FEV1 as a percentage of the expected value is 50%~79%□

GOLD 1: FEV1 as a percentage of predicted value is ≥80%□

1. **Which of the following is not included in assessing COPD patients in subgroups A, B, and E?（Multiple choice）**

history of moderate to severe acute exacerbations in the past 1 year□

mMRC Scale□

CAT Scale□

FEV1/FVC□ (correct answer)

1. **For stable COPD patients whose symptoms are assessed as group B, the preferred drug regimen is?（Multiple choice）**

LABA or LAMA□

ICS+LABA□

LABA+LAMA□ (correct answer)

SABA+SAMA□

SABA□

1. **Which of the following questionnaires can be used to evaluate patients with COPD?（Multiple choice：There may be more than one correct answer.）**

mMRC Scale□ (correct answer)

SGRQ questionnaire□ (correct answer)

CAT Scale□ (correct answer)

ACT Scale□

BODE Index Scale (correct answer)□

1. **Which of the following statements are true for pharmacologic therapy in the stabilized phase of COPD?（Multiple choice：There may be more than one correct answer.）**

bronchodilators are central to symptom control and are usually given in basal amounts to prevent or minimize symptoms□ (correct answer)

Long-term use of oral glucocorticoids is not recommended□ (correct answer)

In patients with a history of acute exacerbations, ICS combined with LABA therapy may improve lung function and reduce the number of acute exacerbations□ (correct answer)

Phlegmolytic agents such as acetylcysteine help patients to eliminate sputum and should be used for a long time□

1. **Which of the following statements is correct regarding the selection of inhaled preparations for patients with COPD?（Multiple choice：There may be more than one correct answer.）**

For patients with inadequate inspiratory flow rate (PIFR <30L/min), DPI is preferred□

For patients with inadequate inspiratory flow rate (PIFR <30L/min), pMDI or SMI is preferred□(correct answer)

For patients with poor hand-to-mouth coordination, it is recommended that a mist reservoir be included with the pMDI□ (correct answer)

For patients with adequate inspiratory flow rates (PIFR ≥ 30 L/min), DPI, pMDI, or SMI is preferred□ (correct answer)

### Ⅱ. Pharmacists' attitude questionnaire on hierarchical management of patients with COPD

1. **Can pharmacists' implementation of hierarchical management of COPD patients improve the capacity and efficiency of pharmacy services and quantify pharmacists' work?**

Strongly agree□ Agree□ Neutral□ Disagree □ Strongly disagree□

1. **Can pharmacists stratify COPD patients based on certain criteria to identify high-risk patients who need more attention?**

Strongly agree□ Agree□ Neutral□ Disagree □ Strongly disagree□

1. **Can pharmacists give higher frequency follow-up and management to patients classified to the high-risk tier?**

Strongly agree□ Agree□ Neutral□ Disagree □ Strongly disagree□

1. **Can pharmacists follow up with patients through cough and asthma pharmacy clinics or by phone?**

Strongly agree□ Agree□ Neutral□ Disagree □ Strongly disagree□

1. **Do you think pharmacists can personalize patient guidance and management based on a comprehensive assessment of information about their disease, medication use, and level of self-management?**

Strongly agree□ Agree□ Neutral□ Disagree □ Strongly disagree□

1. **Is there a need to develop specific implementation criteria for the hierarchical management of patients with COPD to help you better carry out this management in the clinic and meet the need for efficient and high-quality pharmacy services?**

Strongly agree□ Agree□ Neutral□ Disagree □ Strongly disagree□

### Ⅱ. Pharmacists' practice questionnaire on hierarchical management of patients with COPD

1. **Do you perform hierarchical management of patients with COPD?**

Always□ Usually□ Sometimes□ Rarely□ Never□

1. **Do you assess the patient's symptoms, acute exacerbation history, risk factors, comorbidities, and other disease conditions and tailor instruction and management?**

Always□ Usually□ Sometimes□ Rarely□ Never□

1. **Do you assess the patient's medication adherence, inhalation technique, number of medications, and other medications and provide guidance and recommendations accordingly?**

Always□ Usually□ Sometimes□ Rarely□ Never□

1. **Do you assess patients' level of self-management and educate them?**

Always□ Usually□ Sometimes□ Rarely□ Never□

1. **Do you follow up with patients through pharmacy clinics or by phone?**

Always□ Usually□ Sometimes□ Rarely□ Never□

1. **Do you give more attention and follow-up to patients with high-risk levels?**

Always□ Usually□ Sometimes□ Rarely□ Never□
